# Negative Regulation of Cathepsins by β-Amyloid

**DOI:** 10.1523/ENEURO.0258-23.2023

**Published:** 2024-01-09

**Authors:** Brianna Lundin, Anne-Claire Comby, Oksana Berezovska, Masato Maesako

**Affiliations:** MassGeneral Institute for Neurodegenerative Disease, Massachusetts General Hospital, Harvard Medical School, Charlestown 02129, Massachusetts

**Keywords:** Alzheimer's disease, β-amyloid, cathepsin B, cathepsin D, endo-lysosomal pathway

## Abstract

Genome wide association study (GWAS) uncovered Alzheimer's disease (AD) risk genes linked to the endo-lysosomal pathway. This pathway seems to be the gateway of protein aggregates, such as tau and α-synuclein, to the cytoplasm. Furthermore, we and others reported that the amyloid precursor protein (APP) C99 is predominantly processed by γ-secretase in the endo-lysosomal compartments, and β-amyloid (Aβ) peptides are enriched in the same subcellular loci. While the role(s) of APP/Aβ in the endo-lysosomal pathway has not been fully established, a recent study reported that Aβ, in particular Aβ42, inhibits cathepsin D (CTSD) activity. Here, we show using a cell-free in vitro assay that Aβ42 also blocks cathepsin B (CTSB) activity. Furthermore, we uncovered that the autocatalytic processing (i.e., conversion of single chain to heavy/light chains) of CTSB and CTSD is accelerated in APP-deficient cells compared with wild-type controls. Taken together, our findings further support the negative regulation of cathepsins by Aβ.

## Significance Statement

Aβ is generated in endo-lysosomal compartments and secreted to the outside of the cells. While the role of extracellular Aβ has been extensively studied and is considered one of the targets to treat AD, the role(s) of APP/Aβ inside the cells remains elusive. This confirmatory study sheds light on the roles of APP/Aβ in endo-lysosomal compartments.

## Introduction

Mutations in the gene encoding APP and presenilin (PSEN1, PSEN2), the catalytic subunit of γ-secretase, are associated with the early-onset familial forms of Alzheimer's disease (AD; Goate et al., 1991; Levy-Lahad et al., 1995; Sherrington et al., 1995). γ-Secretase cleaves the C-terminal fragment of APP (APP CTFβ, a.k.a. C99), resulting in the generation of β-amyloid (Aβ) peptides (De Strooper et al., 1998; Wolfe et al., 1999). Aβ deposition, the so-called senile plaques, is one of the pathological hallmarks of AD, and several anti-Aβ antibodies recently showed promising results in AD clinical trials (Mintun et al., 2021; Budd Haeberlein et al., 2022; van Dyck et al., 2023). Aβ is predominantly generated in the endo-lysosomal compartments (Haass et al., 1992; Koo and Squazzo, 1994; Sannerud et al., 2016; Maesako et al., 2022) and enriched in the same subcellular loci (Takahashi et al., 2002; Cataldo et al., 2004; Willén et al., 2017; Lee et al., 2022); however, the role(s) of APP/Aβ in the intracellular compartments remains elusive. Of note, two APP homologous proteins exist—amyloid precursor-like protein 1 (APLP1) and APLP2. APP/APLP1 knock-out (KO) mice were viable, while APP/APLP2 and APLP1/APLP2 mice were postnatally lethal, suggesting that the three proteins serve redundant functions and highlighting the essential role of APLP2 in mouse development (Heber et al., 2000).

Cathepsins are proteases responsible for the degradation of various molecules in the endo-lysosomal system (Turk et al., 2012). Cathepsins are known to undergo autocatalysis to divide their single chain into heavy and light chains in the late endosomes and lysosomes (Szulc-Dąbrowska et al., 2020). Functional association between cathepsins and APP/Aβ has been previously reported. For example, cathepsin D (CTSD) is reported to degrade Aβ (McDermott and Gibson, 1996; Gallwitz et al., 2022). On the other hand, a recent study showed that Aβ, Aβ42 in particular, inhibits the activity of CTSD (Suire et al., 2020). Similarly, it is reported that cathepsin B (CTSB) degrades Aβ42 (Mueller-Steiner et al., 2006) as well as APP (Hook et al., 2005), suggesting that Aβ42 and APP can be in proximity to the catalytic site of CTSB. Nevertheless, whether CTSB activity is affected by APP/Aβ remains elusive.

Here we report using an in vitro CTSB activity assay that Aβ42, but not other Aβ species such as Aβ38, Aβ40, and Aβ43, inhibits the activity of CTSB. Strikingly, the autocatalysis of CTSB, which is dependent on CTSB activity, is significantly accelerated in APP-deficient mouse embryonic fibroblast (MEF) cells compared with wild-type (WT) controls. We found that CTSD autocatalysis is also enhanced in APP-lacking cells. Taken together, our findings further support the previous finding that cathepsins are negatively regulated by Aβ.

## Materials and Methods

### Antibodies and reagents

Anti-CTSB antibodies were purchased from Cell Signaling Technology and Abcam. Anti-CTSD antibodies were from Santa Cruz Biotechnology and Abcam. An anti-β-actin antibody was from MilliporeSigma, and an anti-GAPDH antibody was from Cell Signaling Technology. γ-Secretase inhibitor (DAPT) and CTSB inhibitor (Z-Phe-Phe-FMK) were purchased from Abcam, and vehicle control DMSO was from Sigma-Aldrich. Synthetic Aβ38, Aβ40, Aβ42, Aβ43, p3 (i.e., Aβ17-42), and scramble Aβ42 peptides were purchased from rPeptide.

### Cell culture

WT and APP/APLP2 double knock-out (dKO; von Koch et al., 1997) mouse embryonic fibroblasts (MEFs) were cultured in Opti-MEM Reduced Serum Medium (Thermo Fisher Scientific) with 5% FBS (Atlanta Biologicals). The cells were authenticated using STR profiling and monitored for mycoplasma contamination every 2 months.

### Aβ ELISA

WT or APP/APLP2 dKO MEF cells were cultured in serum-free medium for 16 h, the collected medium was centrifuged for 5 min at 600 × *g*, and Aβ levels in the supernatant were measured using the Human/Rat beta-Amyloid (40) and beta-Amyloid (42) ELISA kits (FUJIFILM Wako Chemicals U.S.A.).

### Western blotting

Protein concentration was measured using the BCA Protein Assay Kit (Thermo Fisher Scientific). The concentration normalized samples were mixed with NuPAGE^TM^ LDS Sample Buffer and NuPAGE^TM^ Sample Reducing Agent (Thermo Fisher Scientific). After boiling, the samples were subjected to SDS-PAGE on NuPAGE^TM^ 4–12% Bis-Tris Protein gels using MES running buffer (Thermo Fisher Scientific). Then, proteins on the gels were transferred to nitrocellulose membranes (Thermo Fisher Scientific) using the iBlot^TM^ 2 Gel Transfer Device (Thermo Fisher Scientific) or Bio-Rad Wet electroblotting system (Bio-Rad). The membranes were incubated with primary and corresponding fluorophore-conjugated secondary antibodies, and bands were visualized by the digital imaging system LI-COR Odyssey CLx scanner (LI-COR Biosciences).

### In vitro CTSB activity assay

The cathepsin B inhibitor screening kit (Abcam) was used to determine the effect of Aβ peptides on CTSB activity. Briefly, synthetic Aβ, p3, or scramble peptides were mixed with recombinant CTSB and the 7-amino-4-trifluoromethylcoumarin (AFC)-based fluorescent CTSB peptide substrate at 37°C. AFC fluorescence was measured for 30 min every 5 min (Ex, 355 nm; Em, 482 nm) using the FLUOstar Omega microplate reader (BMG LABTECH), and AFC fluorescence at *t* = 20 min over that at *t* = 10 min was calculated to determine the slope of the reaction. The slope from the “no-peptide” (i.e., recombinant CTSB and AFC fluorescent substrate without peptide) and “no-protease” (i.e., AFC fluorescent substrate only) conditions were set as 0% and 100%, respectively, and relative inhibiting efficiency (%) was calculated.

### Statistical analysis

GraphPad Prism 9 (GraphPad Software) was used to perform statistical analysis. The D’Agostino and Pearson omnibus normality test was used to examine the Gaussian distribution of the data and the variance equality. Unpaired *t* test, Mann–Whitney *U* test, or one-way ANOVA was used to compare the data. At least three independent experiments were performed to ensure the reproducibility of the results.

## Results

**Figure 1.**
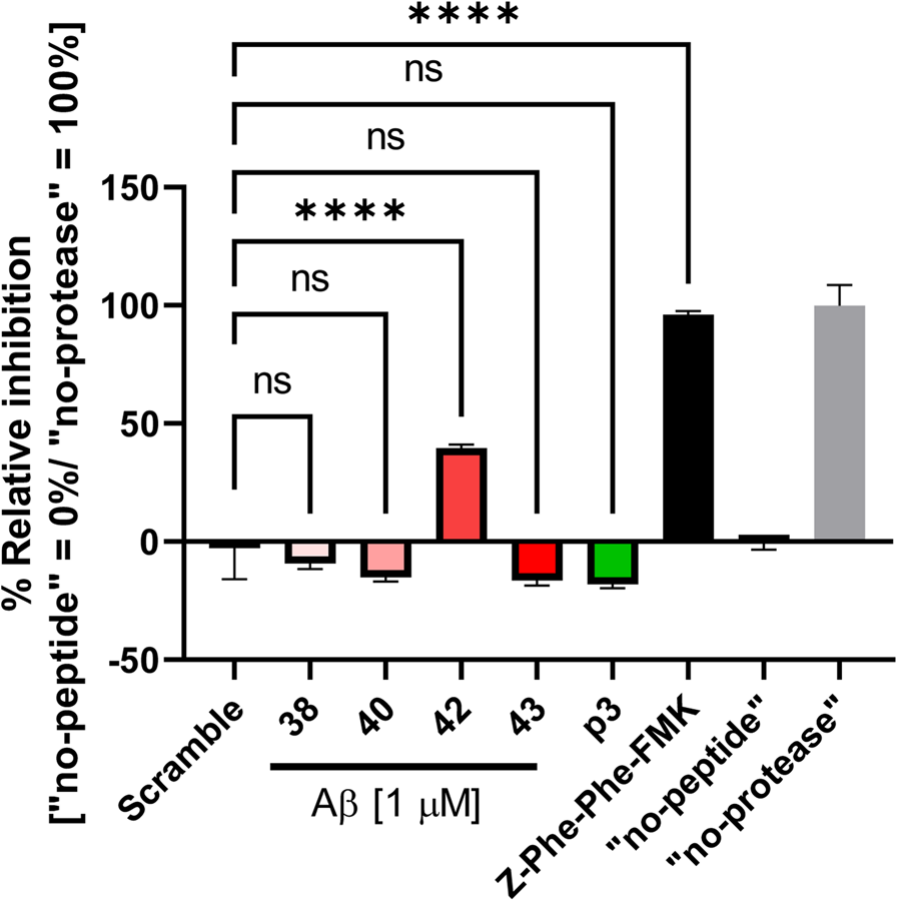
Aβ42 downregulates CTSB activity. AFC-conjugated CTSB substrate and recombinant CTSB were incubated with Aβ38, Aβ40, Aβ42, Aβ43, p3, or scramble Aβ peptides (1 µM). % Relative Inhibition (“no-peptide” and “no-protease” set as 0% and 100%, respectively) is shown. CTSB inhibitor Z-Phe-Phe-FMK (1 µM) was used to ensure the specificity of the in vitro activity assay. *N* = 3, *p < 0.05, ****p < 0.0001, one-way ANOVA. Figure 1-1 is supporting Figure 1.

Various studies, including ours, suggest that Aβ is generated (Haass et al., 1992; Koo and Squazzo, 1994; Sannerud et al., 2016; Maesako et al., 2022) and is enriched in the endo-lysosomal compartments (Takahashi et al., 2002; Cataldo et al., 2004; Willén et al., 2017; Lee et al., 2022). A recent study reported that Aβ peptides, Aβ42 in particular, inhibit CTSD activity (Suire et al., 2020). Here we employed a fluorometric in vitro assay to examine if Aβ peptides may inhibit the activity of CTSB: another cathepsin family protein. In the experiment, 1 µM Aβ38, Aβ40, Aβ42, or Aβ43 was incubated with recombinant CTSB and AFC-conjugated CTSB substrate as the concentration of Aβ is estimated to be at micrometer range in the endo-lysosomal compartments (Hu et al., 2009; Schützmann et al., 2021). Then, the AFC fluorescence at 20 min post incubation was divided by that at 10 min to determine the slope of the reaction (F2/F1 ratio). Lastly, the slope in each group was normalized to those in “no-peptide” and “no-protease” conditions to calculate % Relative CTSB Inhibition. Scramble Aβ42 and p3 (Aβ17-42) peptides (1 µM) were used as the controls of Aβ peptides, and a CTSB inhibitor (1 µM Z-Phe-Phe-FMK) was used to ensure the reliability of the assay. Interestingly, we found that Aβ42, but not Aβ38, Aβ40, and Aβ43, significantly inhibits the activity of CTSB (Fig. 1). We also verified the dose-dependent effect of Aβ42 and found that even 1 nM Aβ42 significantly inhibits CTSB activity (Fig. 1-1).

10.1523/ENEURO.0258-23.2023.f1-1 Figure 1-1 AFC-conjugated CTSB substrate and recombinant CTSB were incubated with different concentrations of Aβ42 (1nM to 5 μM) or scramble Aβ peptides (1 μM). % Relative Inhibition (“no-peptide” and “no-protease” set as 0% and 100%. respectively) is shown. CTSB inhibitor: Z-Phe-Phe-FMK (1 μM) was used to ensure the specificity of the in vitro activity assay. N=3, ****p<0.0001, one-way ANOVA. Download Figure 1-1, DOCX file.

**Figure 2.**
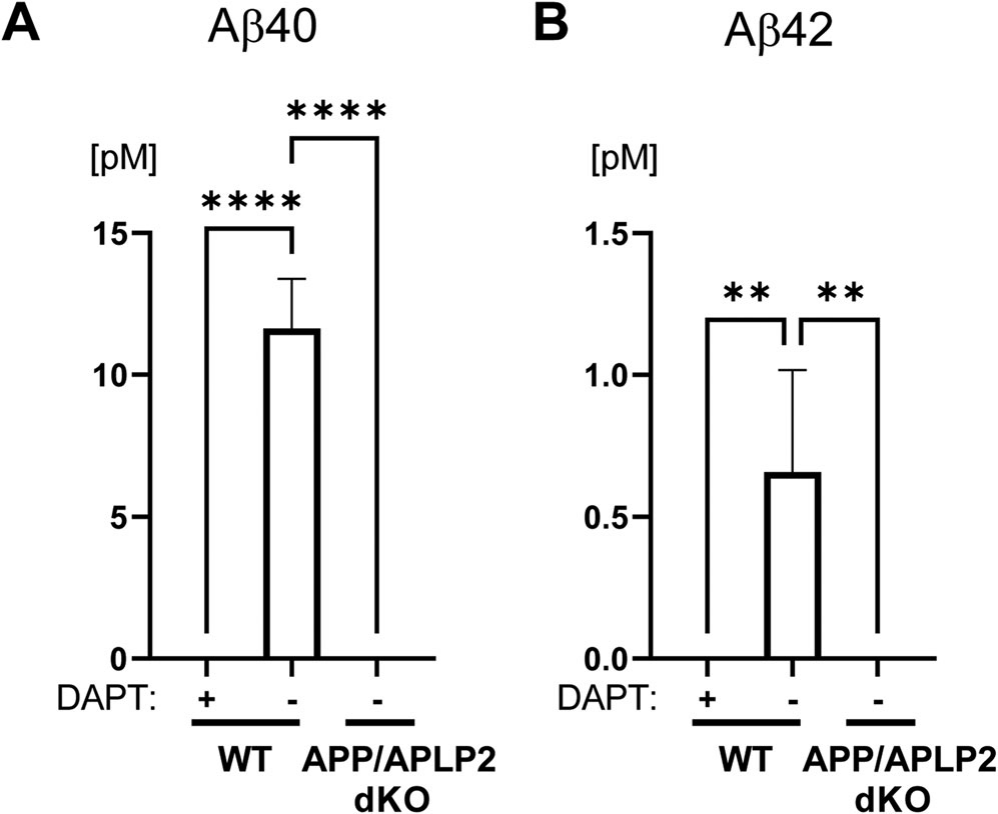
Abolished Aβ generation in APP/APLP2-deficient MEF cells. ***A***, Aβ40 and (***B***) Aβ42 levels in the conditioned medium of APP/APLP2 dKO MEF cells are undetectable as opposed to WT controls. γ-Secretase inhibitor DAPT (1 µM) was used to ensure the specificity of ELISA. *N* = 4, **p < 0.01, ****p < 0.0001, one-way ANOVA.

**Figure 3.**
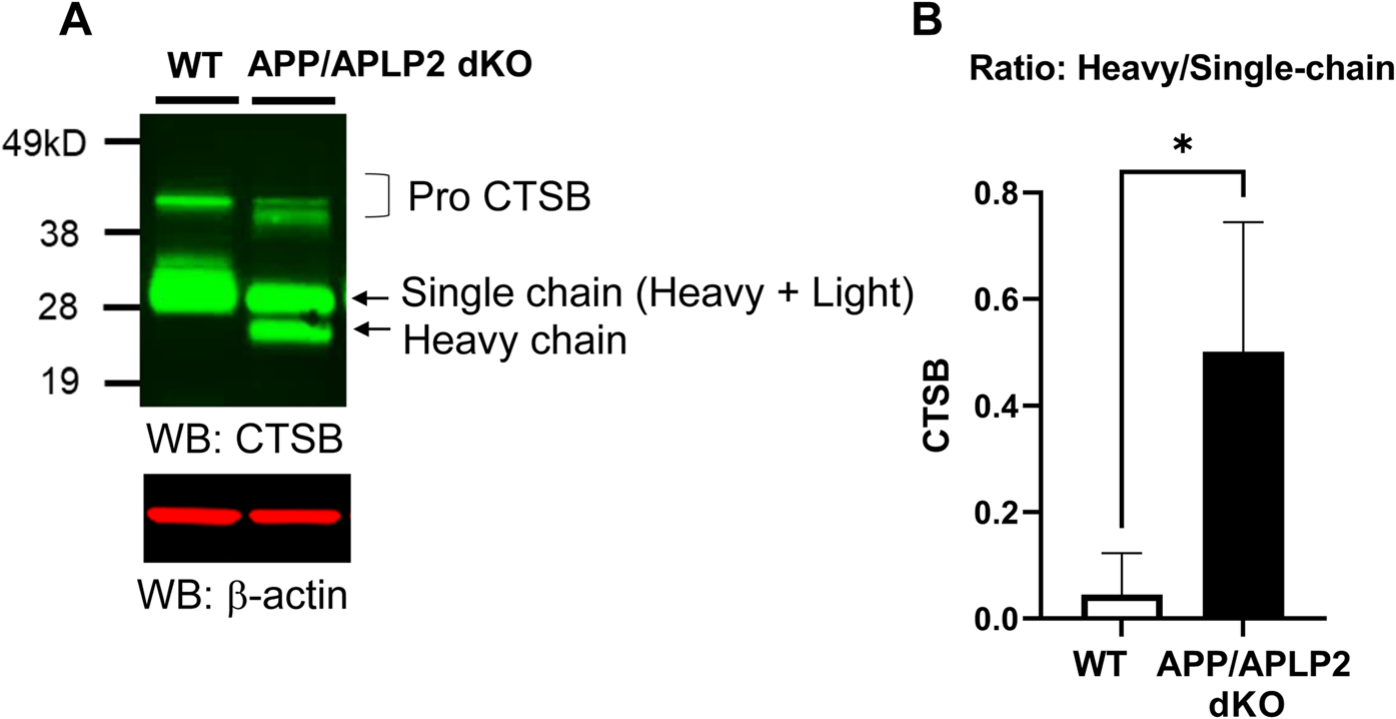
Accelerated CTSB autocatalysis in APP/APLP2-deficient cells. ***A***, A representative Western blot image by detecting with a CTSB antibody, ***B*** shows that the ratio of CTSB heavy chain over single chain is significantly increased in APP/APLP2 knock-out compared with WT MEF cells. *N* = 3 independent experiments, *p < 0.05, Mann–Whitney *U* test. Figures 3-1 and 3-2 are supporting Figure 3.

To validate the finding using a complementary approach, we cultured MEF cells derived from APP/APLP2 dKO mice (von Koch et al., 1997) or WT controls. As expected, we detected Aβ40 and Aβ42 in the conditioned medium of WT MEFs, whereas Aβ40 and Aβ42 levels in the medium of APP/APLP2 dKO MEF cells were not detectable (Fig. 2*A*,*B*). The levels of Aβ40 and Aβ42 in the medium of WT MEF cells treated with a γ-secretase inhibitor DAPT were also undetectable, evidencing the specificity of ELISA. Then, the cell lysates of APP/APLP2 dKO and WT MEF cells were subjected to Western blotting using CTSB antibodies. The bands corresponding to immature (pro) and mature forms (single chain, heavy chain) of CTSB were verified using CTSB and pro-CTSB specific antibodies (Fig. 3-1). Interestingly, we uncovered the level of CTSB heavy chain was increased. In contrast, CTSB single chain was decreased in APP/APLP2 MEF cells (Fig. 3*A*), resulting in significantly higher heavy chain/single chain ratios compared with WT MEF cells (Fig. 3*B*). Of note, CTSB inhibitor Z-Phe-Phe-FMK dose-dependently decreased the ratio of heavy chain over the single chain of CTSB in APP/APLP2 MEF cells (Fig. 3-2), suggesting that CTSB conversion from single chain to heavy/light chains is CTSB activity dependent. These results demonstrate that CTSB autocatalysis is accelerated in APP/APLP2 MEF cells.

10.1523/ENEURO.0258-23.2023.f3-1 Figure 3-1 The immature (i.e., pro-) and mature forms (i.e., single chain and heavy chain) of CTSB are detected in APP/APLP2 dKO cell lysates using the EPR21033 CTSB antibody. On the other hand, the G60 pro-CTSB antibody only detects pro-CTSB around 38 kD, allowing to distinguish between the immature and mature forms of CTSB. Download Figure 3-1, DOCX file.

10.1523/ENEURO.0258-23.2023.f3-2 Figure 3-2 **(A)** Western blotting analysis of APP/APLP2 dKO cells treated with different concentrations of CTSB inhibitor (Z-Phe-Phe-FMK) or vehicle for 16 hours. β-actin was used as a loading control. **(B)** The heavy chain over the single chain ratio was dose-dependently decreased by the treatment with Z-Phe-Phe-FMK, suggesting the CTSB single chain to heave/light chains conversion is dependent on CTSB activity. One-way ANOVA, N = 3 independent experiments, **p < 0.01. Download Figure 3-2, DOCX file.

**Figure 4.**
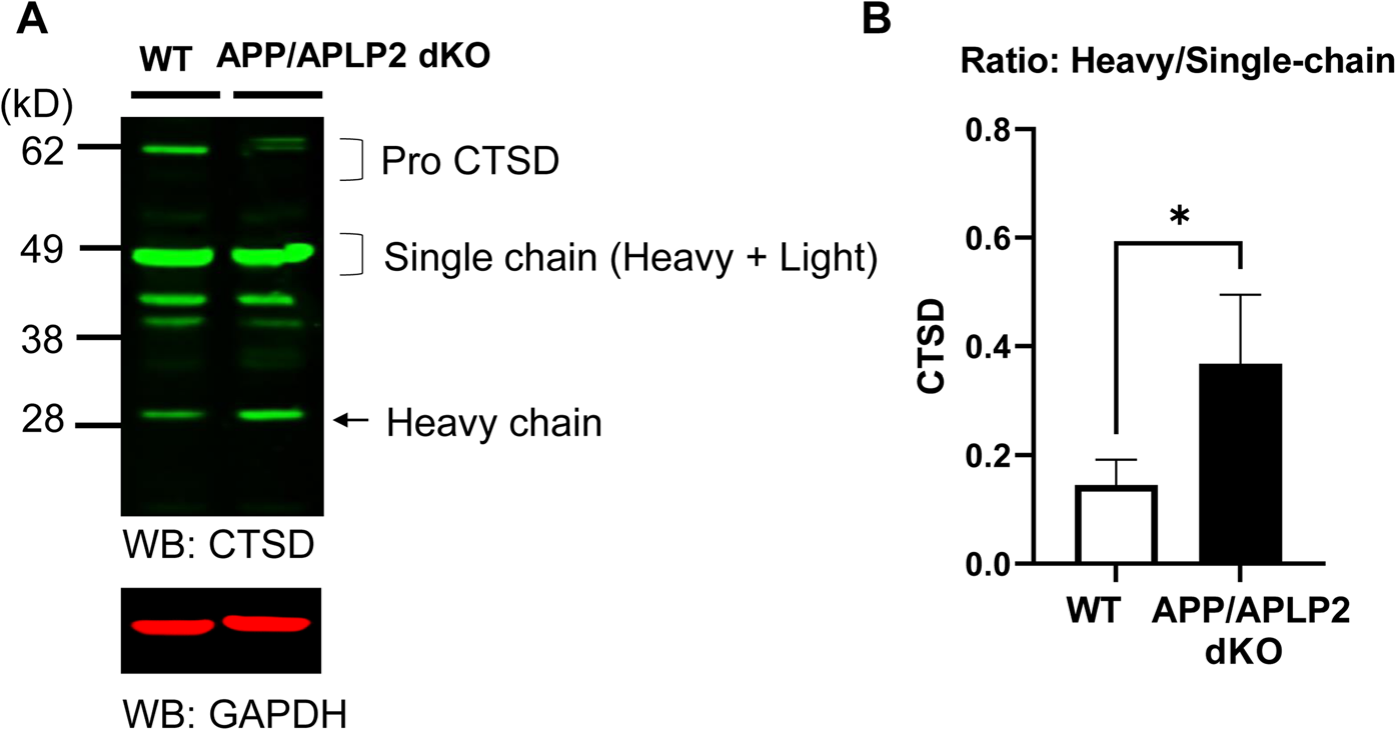
Increased CTSD autocatalysis in APP/APLP2-deficient cells. ***A***, A representative Western blot image by detecting with a CTSD antibody. ***B***, The ratio of CTSD heavy chain over single chain is significantly increased in APP/APLP2 dKO compared with WT MEF cells. *N* = 3 independent experiments, *p < 0.05, Mann–Whitney *U* test. Figure 4-1 is supporting Figure 4.

Lastly, Western blotting membranes of APP/APLP2 and WT MEF cell lysates were probed with CTSD antibodies to examine if CTSD autocatalysis is also increased in APP/APLP2 MEF cells. The bands corresponding to immature (pro) and mature forms of CTSD were verified using CTSD and pro-CTSD specific antibodies (Fig. 4-1). We found that CTSD heavy chain/single chain ratios were also significantly higher in APP/APLP2 dKO MEF cells compared with WT controls (Fig. 4*A*,*B*), suggesting that CTSD autocatalysis is also accelerated in APP/APLP2 dKO compared with WT MEF cells. Collectively, our results suggest that APP/APLP2 and/or Aβ42 negatively regulate the activities of cathepsins.

10.1523/ENEURO.0258-23.2023.f4-1 Figure 4-1 The immature and mature forms (i.e., single chain and heavy chain) of CTSD are detected in APP/APLP2 dKO cell lysates using the D-7 CTSD antibody. However, the EPR24352 pro-CTSD antibody dominantly detects pro-CTSD around 62 kD, enabling to distinguish between immature and mature forms of CTSD. Download Figure 4-1, DOCX file.

## Discussion

Numerous studies demonstrated that APP is processed by β- and γ-secretase in the endo-lysosomal pathway (Haass et al., 1992; Koo and Squazzo, 1994; Huse et al., 2000; Rajendran et al., 2006; Sannerud et al., 2016; Maesako et al., 2022). Our recent studies employing unique molecular sensors (Houser et al., 2020; Maesako et al., 2020) together with multiplexing confocal microscopy enabled “visualizing” that APP C99 is predominantly processed by γ-secretase in late endosomes and lysosomes, and Aβ peptides are enriched in the same subcellular areas (Maesako et al., 2022; McKendell et al., 2022). In addition to Aβ being produced and retained in the endo-lysosomal compartments, previously secreted Aβ can also be taken up by cells and internalized into the acidic compartments (Nagele et al., 2002; Zerbinatti et al., 2006). While a recent study reported that Aβ42 inhibits CTSD activity (Suire et al., 2020), the role of APP/Aβ in the acidic compartments remains unclear. Our study found using a cell-free in vitro system that Aβ42 inhibits CTSB activity (Fig. 1). Furthermore, we uncovered that CTSB and CTSD autocatalysis is significantly accelerated in APP/APLP2-deficient cells compared with that in WT controls (Figs. 3, 4).

Aβ accumulation is the earliest pathological alteration in AD brains. The presence of amyloid plaques accelerates tau propagation and deposition (Pooler et al., 2015; Pontecorvo et al., 2017; Vogel et al., 2020), suggesting that Aβ can lead to progressive tau deposition. However, how Aβ leads to neurofibrillary tau tangles formation remains elusive. Cathepsins, particularly CTSD, are reported to be responsible for the degradation of tau in vitro (Kenessey et al., 1997), in Drosophila and mice models (Khurana et al., 2010). Therefore, this study sought to determine the effect of Aβ on cathepsins in the endo-lysosomal system.

The bidirectional relationship between CTSD and Aβ is well established. For example, it is reported that Aβ is degraded by CTSD (McDermott and Gibson, 1996; Gallwitz et al., 2022). Genetic deletion of CTSD in mice significantly increases insoluble Aβ40 and Aβ42 (Suire et al., 2020). On the other hand, Aβ degradation by CTSD implies that Aβ has access to the CTSD catalytic site, and thus Aβ can impact the activity of CTSD. Indeed, it is reported that Aβ42 significantly inhibits the activity of CTSD (Suire et al., 2020). In agreement with these findings, our study shows that CTSD autocatalysis is significantly accelerated in APP/APLP2-deficient cells that lack Aβ (Fig. 4), suggesting that Aβ, perhaps Aβ42, inhibits CTSD activity in the late endosomes and lysosomes.

A cell-free in vitro assay suggested that Aβ42 blocks CTSB activity (Figs. 1, 1-1). Nevertheless, one challenge is that different lengths of Aβ peptides have distinct aggregation properties, and thus they form oligomers differently during the reaction time in the CTSB assay. Therefore, while it is sure that CTSB activity is significantly inhibited in the presence of Aβ42 peptides, it is difficult to fully determine whether the difference between Aβ42 peptides and the other species is due to the different properties of Aβ monomer or differences in the final concentration of the specific forms.

To further determine the effect of APP/Aβ on CTSB activity “in cells,” we also measured CTSB heavy and single chain levels and calculated CTSB heavy chain over single chain ratios. Strikingly, we found higher CTSB heavy chain/single chain ratios in APP/APLP2-deficient cells (Fig. 3). As expected, a potent CTSB inhibitor decreased CTSB heavy chain while increasing single chain levels in a dose-dependent manner (Fig. 3-2), suggesting that CTSB single chain to heavy and light chains conversion is dependent on CTSB activity. Therefore, the lower CTSB heavy chain/single chain ratios in WT MEFs compared with APP/APLP2-deficient cells could be indicative of decreased CTSB activity in WT MEFs, and APP/Aβ (and/or APLP2/APLP2β) blocks the activity of CTSB in late endosomes and lysosomes. Nevertheless, other indirect processes could account for the results since we cannot rule out the possibility that other proteases are involved in the conversion of CTSB single chain to heavy and light chains. Of note, the difference in the heavy/single chain ratio between APP/APLP2 dKO and control WT MEFs is much more significant in CTSB than in CTSD (Figs. 3, 4). This could be because not only Aβ but also APP may negatively regulate CTSB activity. Indeed, it is reported that CTSB degrades both Aβ (Mueller-Steiner et al., 2006) and APP (Hook et al., 2005).

In summary, we found that CTSB and CTSD autocatalytic processing is accelerated in APP-deficient cells compared with WT controls. Furthermore, in addition to reported Aβ42 effect on CTSD, we show CTSB activity is also inhibited in the presence of Aβ42. These results suggest that APP (APLP2) and/or Aβ42 negatively regulate cathepsins, which can be one of the molecular mechanisms by which tau pathology is accelerated in the presence of Aβ.

## Synthesis

Reviewing Editor: Karl Herrup, University of Pittsburgh School of Medicine Decisions are customarily a result of the Reviewing Editor and the peer reviewers coming together and discussing their recommendations until a consensus is reached. When revisions are invited, a fact-based synthesis statement explaining their decision and outlining what is needed to prepare a revision will be listed below. The following reviewer(s) agreed to reveal their identity: NONE.

The authors are to be commended for a thorough and thoughtful response to the previous comments. The revised manuscript is a strong addition to the AD literature.
