# Supplementary material for: Negative Regulation of Cathepsins by β-Amyloid
Source: eNeuro. 2024 Jan 9;11(1):ENEURO.0258-23.2023. doi: 10.1523/ENEURO.0258-23.2023 (PMC10849021; doi:10.1523/ENEURO.0258-23.2023)
Supplement: Figure 1-1 — AFC-conjugated CTSB substrate and recombinant CTSB were incubated with different concentrations of Aβ42 (1nM to 5 μM) or scramble Aβ peptides (1 μM). % Relative Inhibition (“no-peptide” and “no-protease” set as 0% and 100%. respectively) is shown. CTSB inhibitor: Z-Phe-Phe-FMK (1 μM) was used to ensure the specificity of the in vitro activity assay. N=3, ****p<0.0001, one-way ANOVA. Download Figure 1-1, DOCX file. [file eneuro-11-ENEURO.0258-23.2023-s001.docx]

**Extended Figure 1-1**
