# Supplementary figures and images for: Negative Regulation of Cathepsins by β-Amyloid
Source: eNeuro. 2024 Jan 9;11(1):ENEURO.0258-23.2023. doi: 10.1523/ENEURO.0258-23.2023 (PMC10849021; doi:10.1523/ENEURO.0258-23.2023)

**Extended Figure 3-1**

**
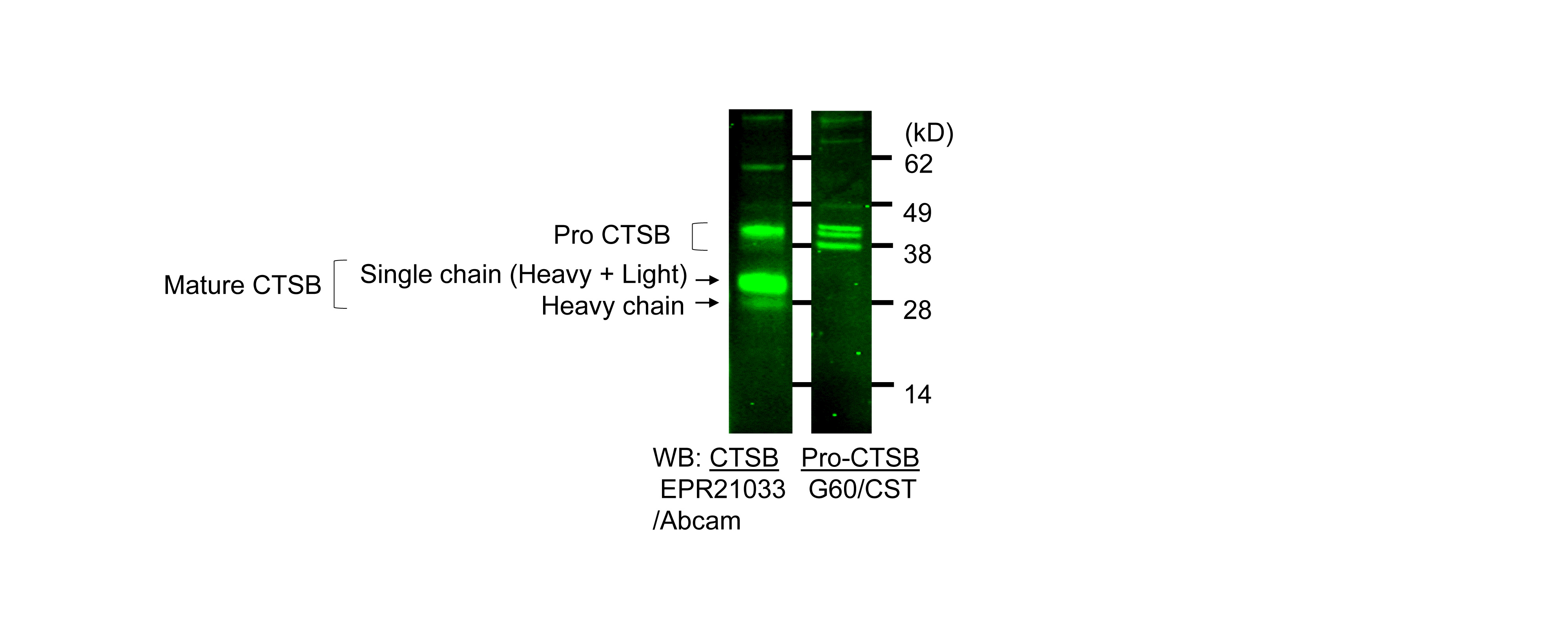
**

Supplement: Figure 3-1 — The immature (i.e., pro-) and mature forms (i.e., single chain and heavy chain) of CTSB are detected in APP/APLP2 dKO cell lysates using the EPR21033 CTSB antibody. On the other hand, the G60 pro-CTSB antibody only detects pro-CTSB around 38 kD, allowing to distinguish between the immature and mature forms of CTSB. Download Figure 3-1, DOCX file. [file eneuro-11-ENEURO.0258-23.2023-s002.docx]

**Extended Figure 3-2**


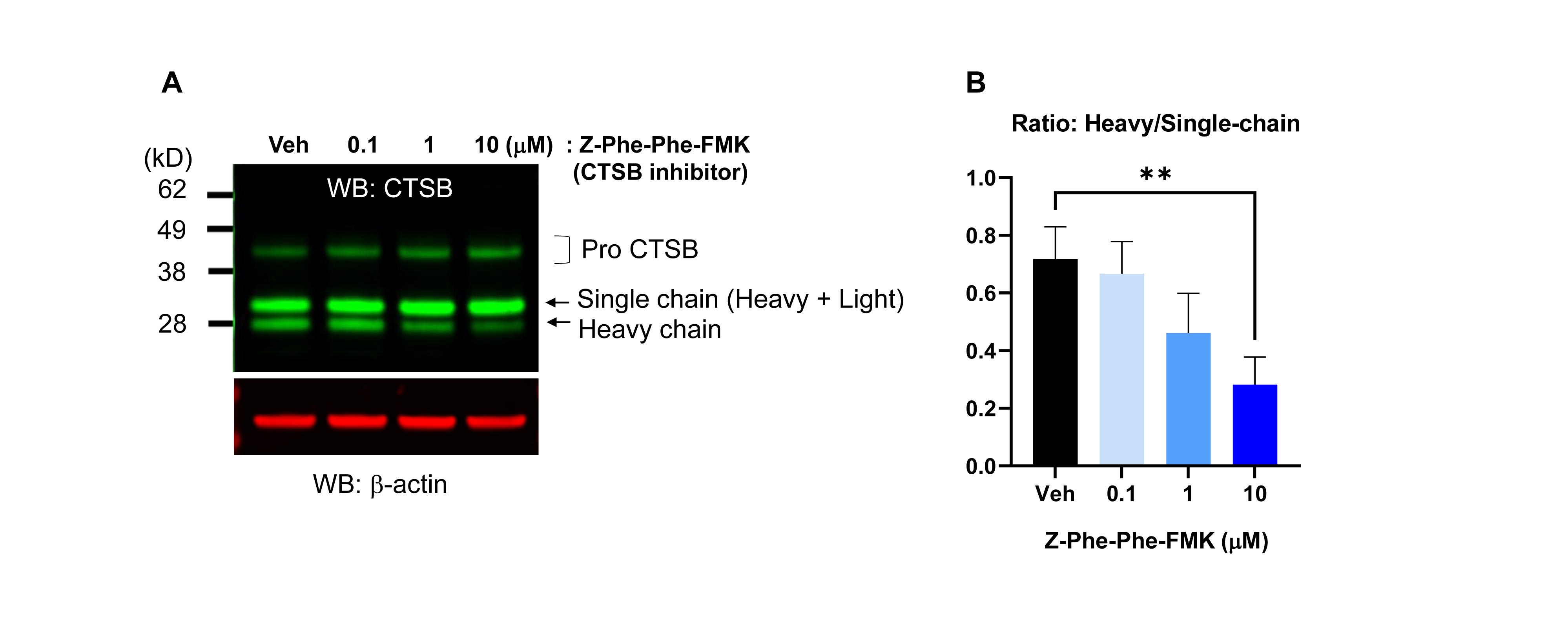

Supplement: Figure 3-2 — (A) Western blotting analysis of APP/APLP2 dKO cells treated with different concentrations of CTSB inhibitor (Z-Phe-Phe-FMK) or vehicle for 16 hours. β-actin was used as a loading control. (B) The heavy chain over the single chain ratio was dose-dependently decreased by the treatment with Z-Phe-Phe-FMK, suggesting the CTSB single chain to heave/light chains conversion is dependent on CTSB activity. One-way ANOVA, N = 3 independent experiments, **p < 0.01. Download Figure 3-2, DOCX file. [file eneuro-11-ENEURO.0258-23.2023-s003.docx]

**Extended Figure 4-1**


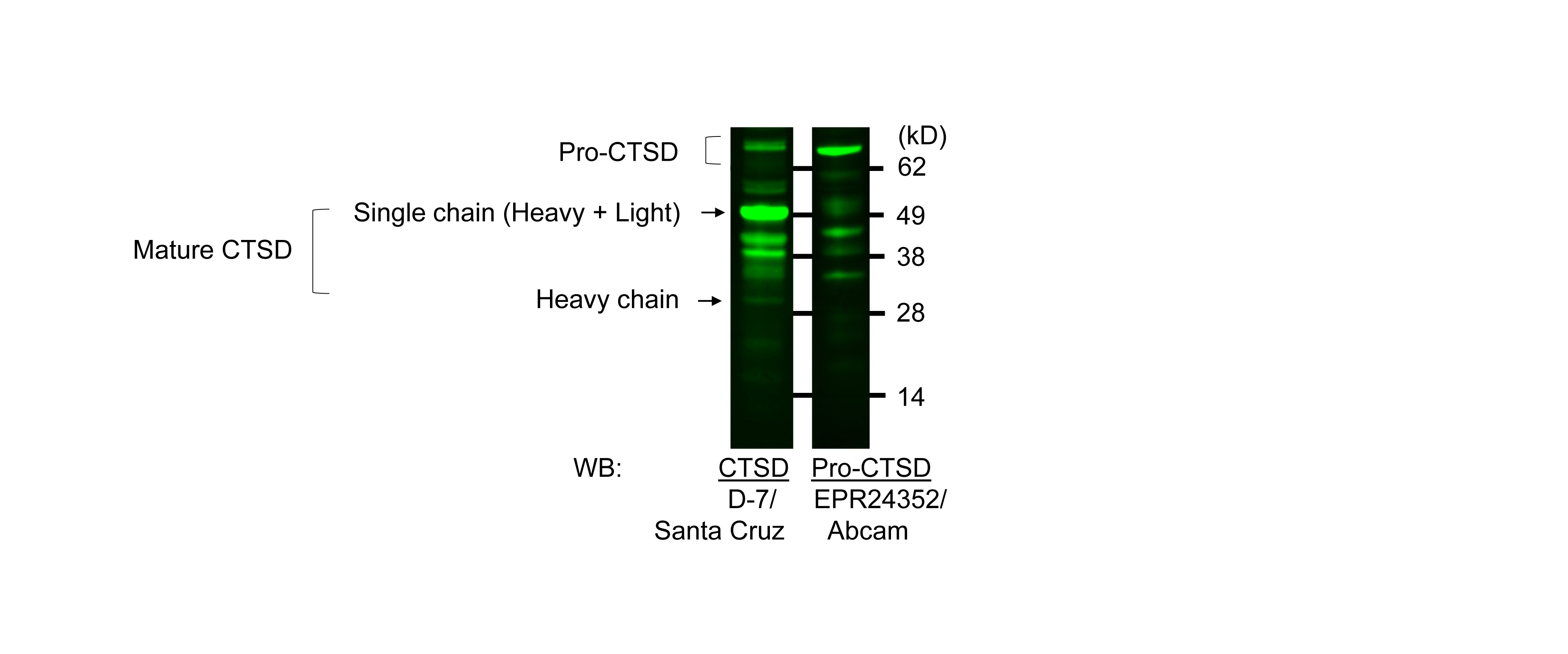

Supplement: Figure 4-1 — The immature and mature forms (i.e., single chain and heavy chain) of CTSD are detected in APP/APLP2 dKO cell lysates using the D-7 CTSD antibody. However, the EPR24352 pro-CTSD antibody dominantly detects pro-CTSD around 62 kD, enabling to distinguish between immature and mature forms of CTSD. Download Figure 4-1, DOCX file. [file eneuro-11-ENEURO.0258-23.2023-s004.docx]
